# Statistical Reporting Quality in Physical Medicine and Rehabilitation Journals: A Comparative Analysis of SCI-E and TR Indexed Publications

**DOI:** 10.5152/eurasianjmed.2026.261511

**Published:** 2026-08-25

**Authors:** Tuğba ahbaz, Gülsev Özen Yorgancıgil, Ahmet Dirican

**Affiliations:** 1 Department of Physical Medicine and Rehabilitation, İstanbul Beykent University Faculty of Medicine, İstanbul, Türkiye; 2 Department of Biostatistics, İstanbul - Cerrahpaşa University Faculty of Medicine, İstanbul, Türkiye; 3 Department of Pharmacology, İstanbul University Faculty of Pharmacy, İstanbul, Türkiye

**Keywords:** Biomedical research, physical medicine and rehabilitation, research methodology, reporting standards, statistical analysis

## Abstract

**Background:**

To evaluate statistical reporting quality and methodological errors in clinical research articles published in physical medicine and rehabilitation (PM&R) journals indexed in Science Citation Index Expanded (SCI-E) and Turkish National Citation Index (TR Dizin).

**Methods:**

This cross-sectional methodological study analyzed original clinical research articles published between January 2024 and January 2025 in 18 PM&R journals indexed in SCI-E and TR Dizin. Articles were evaluated using predefined criteria based on Consolidated Standards of Reporting Trials (CONSORT) statement, the Strengthening the Reporting of Observational Studies in Epidemiology (STROBE) statement, and the Statistical Analyses and Methods in the Published Literature (SAMPL) guidelines. Statistical reporting practices, methodological indicators, and transparency indicators were assessed. Comparisons between journal groups were performed using Pearson’s chi-square or Fisher’s exact tests.

**Results:**

A total of 433 articles were included, of which 358 (82.7%) were published in SCI-E journals and 75 (17.3%) in TR Dizin journals. Mean ± SD was reported in 93.1% of articles, whereas median-based statistics were reported in 47.3%. Normality testing was performed in 72.7% of studies. Effect size reporting was observed in 51.0% of articles. SCI-E journals reported effect sizes more frequently than TR Dizin journals (53.1% vs. 36.0%, *P* = .004). *P* reporting errors were more common in TR Dizin journals (21.3% vs. 8.9%, *P* = .002).

**Conclusion:**

Statistical reporting deficiencies remain common in PM&R clinical research articles. Although SCI-E indexed journals demonstrate relatively better adherence to recommended statistical practices, important methodological shortcomings persist in both indexing groups. Strengthening statistical education and improving editorial oversight may enhance the methodological quality of rehabilitation research.

**Main Points**

- Statistical reporting errors are common in biomedical research.
- Provides updated evidence on statistical reporting in physical medicine and rehabilitation (PM&R) journals.
- Highlights the need for stronger statistical review in journals.

## Introduction

Statistical analysis constitutes a cornerstone of biomedical research, underpinning the validity, reliability, and reproducibility of scientific findings. In clinical research, appropriate methodological planning and transparent statistical reporting are essential for safeguarding evidence-based medical practice and promoting statistical literacy among clinicians and researchers.^1^ In the field of physical medicine and rehabilitation (PM&R), where patient populations are heterogeneous and clinical outcomes are frequently multifactorial, methodological rigor is particularly critical.^2^

Despite advances in statistical methodology and the widespread availability of analytical software, statistical errors remain prevalent in biomedical publications. Methodological evaluations across biomedical disciplines have consistently documented substantial rates of statistical errors and reporting deficiencies in published research. For example, previous studies have shown that a considerable proportion of biomedical articles contain at least one statistical error, often related to inappropriate statistical test selection, inadequate data summarization, or incomplete reporting of analytical procedures.^3^^-^^8^

Among the most frequently reported problems are the use of parametric tests without verification of distributional assumptions, inadequate control of multiple comparisons, and insufficient reporting of post-hoc analyses.^3^^,^^6^ Furthermore, the overemphasis on dichotomous *P* thresholds has been widely criticized in the statistical reform literature, with increasing calls to move beyond sole reliance on “*P* < .05” and to incorporate effect sizes and measures of precision into interpretation, particularly given the recognized disparity between statistical and clinical significance.^9^^-^^12^ Inadequate statistical reporting may contribute to research waste, limit comparability across studies, and hinder effective translation of findings into clinical practice.^13^ Poor statistical reporting may not only compromise the validity of scientific findings but may also influence clinical interpretation and evidence-based decision making.

To enhance methodological transparency and reporting quality, international initiatives such as Consolidated Standards of Reporting Trials (CONSORT) statement, the Strengthening the Reporting of Observational Studies in Epidemiology (STROBE) statement, and the Statistical Analyses and Methods in the Published Literature (SAMPL) have established structured recommendations for clear description of study design, statistical methods, and presentation of results.^14^^-^^16^ These guidelines emphasize explicit identification and justification of statistical procedures, comprehensive reporting of outcomes, and avoidance of misleading interpretations. Nevertheless, empirical investigations continue to demonstrate incomplete adherence to these standards across medical disciplines, and recent evidence highlights the need for more rigorous implementation of SAMPL recommendations in biomedical journals.^4^^,^^17^

While statistical deficiencies have been widely examined in general biomedical research, methodological evaluations in fields such as radiology, dentistry, and family medicine have also reported substantial levels of statistical errors and reporting deficiencies in published articles.^18^^,^^19^ However, specialty-specific evaluations focusing on rehabilitation research remain comparatively limited. Although previous work has described trends in research design and statistical methods within rehabilitation journals,^2^ systematic analyses focusing specifically on the prevalence and patterns of statistical errors in PM&R publications are scarce.

Moreover, methodological rigor may vary according to journal indexing status. Journals indexed in Science Citation Index Expanded (SCI-E) are generally regarded as applying stricter editorial and peer-review standards than national indexing systems such as Turkish National Citation Index (TR Dizin). However, empirical evidence examining whether such differences are reflected in measurable variations in statistical reporting practices within PM&R literature remains limited.^4^^,^^19^ Understanding these potential differences may provide insight into how editorial policies and indexing standards influence methodological quality.

Therefore, the present study aimed to systematically evaluate statistical reporting quality and methodological errors in clinical research articles published in PM&R journals indexed in SCI-E and TR Dizin between January 2024 and January 2025. By analyzing 433 articles using predefined error criteria, this study aims to (1) determine the prevalence and distribution of common statistical errors and (2) compare methodological and reporting practices according to journal indexing type. By providing specialty-specific empirical data, this study seeks to support improvements in statistical education, editorial oversight, and peer-review processes within rehabilitation research.

## Material and Methods

This study was designed as a cross-sectional methodological analysis aimed at systematically identifying, classifying, and comparing statistical errors in clinical research articles published in the field of physical medicine and rehabilitation (PM&R). The primary objective was to determine the prevalence and characteristics of statistical errors and to evaluate potential differences in statistical reporting quality between international journals indexed in the Science Citation Index Expanded (SCI-E) and national journals indexed in the TR Dizin.

The study population consisted of original clinical research articles published between January 2024 and January 2025 in PM&R journals indexed in SCI-E or TR Dizin. PM&R journals were identified through the Web of Science Core Collection database using the keywords “physical medicine” and “rehabilitation” Journals indexed in the SCI-E category were screened according to the order generated by the database search results, and the first 15 eligible journals were included in the study.

For national journals, the TR Dizin database was searched using the Turkish keywords “fiziksel tıp” and “rehabilitasyon” and three journals meeting the inclusion criteria were identified.

Clinical research articles that met the predefined eligibility criteria, were accessible in full text, and were published within the specified time frame were included in the screening process. In journals with a limited number of eligible publications, all eligible full-text articles were included in the analysis. In journals with a high volume of eligible publications, a subset of articles was randomly selected using the RAND function in Microsoft Excel in order to maintain comparable representation across journal groups.

Only original clinical investigations were included. Review articles, systematic reviews, meta-analyses, case reports, case series, letters to the editor, technical notes, and editorials were excluded to ensure methodological homogeneity and to focus exclusively on original clinical research. After applying the eligibility criteria, a total of 433 articles were included in the final analysis, of which 358 (82.7%) were published in SCI-E indexed journals and 75 (17.3%) in TR Dizin indexed journals.

To evaluate statistical quality, a structured assessment framework was developed based on internationally recognized reporting standards, including the CONSORT statement,^15^ STROBE statement,^14^ and SAMPL guidelines.^16^ These guidelines emphasize transparent reporting of study design, statistical methods, and results interpretation.^14^^-^^16^ The evaluation criteria were also informed by previously documented categories of common statistical errors in biomedical research.^3^^-^^5^

All articles were independently reviewed by 2 researchers with experience in research methodology using a predefined evaluation framework. The predefined criteria were applied consistently across all articles to ensure methodological standardization and minimize subjective interpretation. When discrepancies occurred between reviewers, the cases were discussed among the authors until consensus was reached. The classification of inappropriate statistical test use was based exclusively on the statistical methods and data characteristics reported within the articles. No re-analysis or raw data simulation was performed. Statistical procedures were evaluated according to commonly accepted biostatistical principles and relevant reporting guidelines, including CONSORT, STROBE, and SAMPL.

The evaluation encompassed several domains. First, descriptive statistics were assessed to determine whether continuous variables are appropriately reported as mean ± standard deviation (SD), median with interquartile range (IQR), and minimum–maximum values according to data distribution, and whether categorical variables are correctly presented as frequencies and percentages. Second, statistical test selection and application were examined, including verification of normality assumptions prior to parametric testing and appropriate use of nonparametric alternatives when assumptions were violated. Third, inferential procedures were evaluated, particularly the use of post-hoc analyses in multi-group comparisons and the implementation of multiple comparison corrections (e.g., Bonferroni or related procedures) to control type I error.^6^ Fourth, statistical reporting practices were reviewed, including the style of *P* presentation (closed versus open form), reporting of effect size measures and confidence intervals in accordance with contemporary statistical reform recommendations.^9^^-^^12^ documentation of power analysis and sample size justification, and consistency between results reported in text, tables, and figures. Finally, transparency indicators were assessed, including specification of statistical software and version, declaration of ethical approval, and statements regarding data confidentiality and informed consent.

This study used ChatGPT (OpenAI) and IBM SPSS Statistics version 25 (IBM Corp., Armonk, NY, USA) to improve language clarity and edit grammar during the preparation of the manuscript. However, all content of the study has been reviewed and edited by the authors following the use of AI/LLM tools.

### Statistical Analysis

All extracted data were entered into a structured database and analyzed using IBM SPSS Statistics, version 25 (IBM Corp., Armonk, NY, USA). Descriptive statistics were calculated to determine the frequency and percentage of each error category. Comparative analyses between SCI-E and TR Dizin indexed journals were conducted using Pearson’s chi-square test, with Fisher’s exact test applied when expected cell counts were less than 5. All statistical analyses were two-tailed, and statistical significance was set at *P* < .05.

This study was based exclusively on the analysis of publicly available published articles and did not involve human participants, patient data, or identifiable personal information. Therefore, ethical approval and informed consent were not required.

## Results

A total of 433 original clinical research articles published between January 2024 and January 2025 were included in the analysis. Among these articles, 358 (82.7%) were published in SCI-E indexed journals, whereas 75 (17.3%) were published in TR Dizin indexed journals.

### Overall Statistical Reporting Quality

The overall distribution of statistical reporting practices and methodological indicators across the evaluated articles is presented in Table 1.

Descriptive statistics were reported in the majority of studies. Specifically, 403 articles (93.1%) reported mean ± standard deviation, whereas 205 articles (47.3%) reported median, interquartile range, or minimum–maximum values. However, 171 articles (39.5%) did not report median-based descriptive statistics, and in 57 articles (13.2%) such reporting was considered not applicable.

Regarding statistical test reporting, 424 articles (97.9%) clearly defined the statistical tests used, while 9 articles (2.1%) failed to explicitly identify the applied tests. Incorrect naming of statistical tests was observed in 8 articles (1.8%), whereas 3 articles (0.7%) reported statistical tests that were not actually applied in the analysis. In addition, 16 articles (3.7%) were identified as using inappropriate statistical tests relative to the study design or data structure.

### *P-*Value Reporting Errors

Several issues in statistical reporting practices were identified. Closed-form *P* presentation (e.g., *P* < .05) was observed in 214 articles (49.4%), while 219 articles (50.6%) did not report *P* in closed form. Other reporting errors included *P*-value reporting errors in 48 articles (11.1%), statistical notation errors (e.g., non-italicized *P* values, incorrect statistical symbols, or inconsistent notation styles) in 43 articles (9.9%), and inconsistencies between text, tables, and figures in 49 articles (11.3%). Additionally, 24 articles (5.5%) did not report important *P* despite presenting statistical comparisons, and *P*-value inconsistencies were detected in 9 articles (2.1%).

### Methodological Practices

Several methodological indicators related to statistical planning and analysis were also evaluated. Appropriate normality assessment was performed in 315 articles (72.7%), whereas 86 articles (19.9%) applied statistical tests without evaluating the distribution of the data. In 32 articles (7.4%), normality testing was considered not applicable. Sample size calculation was reported in 243 articles (56.1%), whereas 174 articles (40.2%) did not report any sample size estimation. Post-hoc analyses were conducted in 138 studies (31.9%), while 40 studies (9.2%) did not perform post-hoc testing despite involving multiple group comparisons. In 255 studies (58.9%), post-hoc analysis was not applicable. Multiple comparison correction procedures were reported in 120 studies (27.7%), whereas 58 studies (13.4%) conducted multiple comparisons without applying any correction method. In 255 studies (58.9%), correction procedures were considered not applicable. Effect size measures were reported in 221 articles (51.0%), whereas 212 articles (49.0%) did not report effect size estimates.

### Transparency Indicators

Transparency indicators related to reporting practices were also assessed. Statistical software used for analysis was reported in 407 articles (94.0%), while 26 articles (6.0%) did not specify the statistical software used. Ethical approval statements were present in 412 articles (95.1%), whereas 3 articles (0.7%) did not report ethical approval. In another 3 articles (0.7%), ethical approval was considered not applicable. Statements regarding data confidentiality or informed consent were reported in 330 articles (76.2%), while 69 articles (15.9%) did not include such statements, and 19 articles (4.4%) were classified as not applicable.

### Comparison Between SCI-E and TR Dizin Indexed Journals

A comparison of statistical reporting practices between SCI-E and TR Dizin indexed journals is presented in Table 2.

No significant differences were observed between the two indexing groups regarding the reporting of mean ± SD (*P* = .604) or median-based descriptive statistics (*P* = .380). However, several methodological indicators showed significant differences between indexing groups. Appropriate normality assessment was reported more frequently in TR Dizin journals (88.0%) compared with SCI-E journals (69.6%) (*P* = .003).

Similarly, multiple comparison correction procedures were more frequently reported in SCI-E journals (27.4%) than in TR Dizin journals (20.0%) (*P* = .019). Effect size reporting was also significantly more common in SCI-E indexed articles (53.1%) compared with TR Dizin indexed articles (36.0%) (*P* = .004).

Regarding *P*-value reporting practices, closed-form *P*-value presentation was significantly more frequent in TR Dizin journals (73.3%) than in SCI-E journals (44.4%) (*P* < .001). In contrast, *P*-value reporting errors were more common in TR Dizin journals (21.3%) compared with SCI-E journals (8.9%) (*P* = .002).

No statistically significant differences were observed between indexing groups regarding incorrect statistical test naming, inappropriate test application, *P*-value inconsistency, statistical notation errors, or inconsistencies between text, tables, and figures.

Finally, ethical approval reporting was similarly high in both indexing groups (SCI-E: 99.1%; TR: 100%; *P* = 1.000). However, data confidentiality statements were significantly more frequent in SCI-E indexed journals (86.5%) compared with TR indexed journals (61.0%) (*χ*² = 21.68, df = 1, *P* < .001).

## Discussion

The present study systematically evaluated statistical reporting quality and methodological errors in clinical research articles published in PM&R journals indexed in SCI-E and TR Dizin between 2024 and 2025. By analyzing a total of 433 articles, our findings demonstrate that statistical errors and reporting deficiencies remain common across PM&R publications. Importantly, several methodological indicators—including the reporting of descriptive statistics, verification of statistical assumptions, effect size reporting, and transparency indicators—differed significantly between international and national journals. These findings are consistent with previous investigations demonstrating persistent deficiencies in statistical reporting across biomedical research fields.^3^^,^^4^

One of the principal findings of this study was the inadequate reporting of descriptive statistics. Although mean ± SD was frequently reported, the presentation of median values and dispersion measures appropriate for non-normally distributed data (e.g., interquartile range or minimum–maximum values) was considerably less common. Proper reporting of descriptive statistics is essential because inappropriate summary measures may distort the distribution of the data and lead to misleading interpretations.^4^^,^^20^ Previous evaluations of biomedical literature have similarly shown that descriptive statistics are often selected without adequate consideration of distributional assumptions.^3^^,^^21^ The higher adherence observed in SCI-E journals may reflect stricter editorial policies and greater familiarity with international reporting standards such as CONSORT, STROBE, and SAMPL.^14^^,^^15^^,^^22^

Another important methodological issue identified in this study was the inappropriate use of parametric statistical tests without verification of underlying assumptions. A substantial proportion of the evaluated articles applied parametric tests without reporting normality testing. This issue has been documented across multiple biomedical disciplines, including radiology, nursing, and psychology journals.^2^^,^^4^ Failure to assess statistical assumptions undermines the validity of inferential conclusions and increases the risk of inaccurate statistical inference. The SAMPL guidelines emphasize that authors should clearly report the assumptions underlying statistical tests and justify the choice of statistical methods used in the analysis.^15^ Interestingly, although TR Dizin journals demonstrated higher rates of reported normality assessment, they also exhibited significantly higher frequencies of *P*-value reporting errors. This finding may suggest that formal adherence to reporting requirements alone does not necessarily ensure appropriate statistical interpretation or methodological rigor.

The analysis also revealed deficiencies in the implementation of post-hoc analyses and multiple comparison corrections. Among studies involving comparisons between more than two groups, correction procedures were not consistently applied. This omission is methodologically problematic because multiple testing substantially increases the probability of type I errors. Previous research has demonstrated that failure to apply appropriate multiple comparison corrections remains common in clinical research.^3^^,^^6^ These findings suggest that the problem persists even in recent publications, indicating that awareness of multiplicity adjustments remains insufficient.

In terms of statistical reporting practices, the continued use of open-form *P* (e.g., *P* < .05) rather than exact *P* was another common issue identified in our analysis. Modern statistical reporting recommendations strongly encourage the presentation of exact *P* in order to allow readers to interpret the strength of evidence more precisely.^22^^,^^23^ Furthermore, the American Statistical Association has emphasized that reliance solely on arbitrary significance thresholds can lead to misinterpretation of research findings and should be complemented by additional measures such as effect sizes and confidence intervals.^10^

Perhaps the most notable finding of this study was the low rate of effect size reporting and sample size justification. Effect sizes were reported in only a small proportion of the evaluated articles, and power analyses were similarly uncommon. These findings align with previous systematic reviews indicating that effect size reporting remains insufficient in many areas of biomedical research.^21^^-^^23^ Reporting effect sizes is essential because statistical significance alone does not convey the magnitude or clinical importance of study findings. The CONSORT and SAMPL guidelines explicitly recommend that authors report both effect size estimates and confidence intervals to facilitate interpretation and reproducibility.^14^^,^^15^

Although sample size calculation reporting was evaluated in the present study, the specific methodological approaches used for effect size determination (e.g., literature-based estimates, pilot data, or conventional effect size assumptions such as Cohen’s criteria) were not systematically classified. Since effect size selection remains a debated and context-dependent issue in biomedical research, future methodological investigations focusing specifically on sample size planning strategies may provide additional insight into the statistical rigor of PM&R publications.

Transparency indicators related to statistical methodology and research ethics also revealed important patterns. While most articles specified the statistical software used, version numbers were frequently omitted. Reporting software versions is recommended because computational algorithms and default procedures may vary between software releases, potentially affecting reproducibility. Ethical approval statements were reported at high rates in both groups of journals, reflecting improved awareness of research ethics requirements in biomedical research. However, data confidentiality statements were significantly less frequent in TR Dizin journals, suggesting inconsistencies in transparency reporting. Similar shortcomings in transparency indicators have been reported in previous methodological assessments of biomedical literature.^22^^,^^24^

Several factors may explain the observed differences between SCI-E and TR Dizin journals. International journals typically apply more rigorous editorial standards, often involve statistical reviewers during the peer-review process, and require stricter adherence to established reporting guidelines. In contrast, national journals may face challenges related to limited reviewer expertise, variations in methodological training, and inconsistent enforcement of reporting standards. Additionally, language barriers and limited access to formal biostatistics support may contribute to the persistence of statistical errors in certain research environments.

The implications of these findings are important for the PM&R research community. Inadequate statistical practices may compromise the reliability and reproducibility of scientific findings and potentially influence clinical decision-making. Improving statistical literacy among clinicians and researchers is therefore essential. Educational initiatives focusing on appropriate test selection, effect size interpretation, and transparent reporting practices could substantially improve the methodological quality of future studies. In addition, journal editors and peer reviewers play a critical role in ensuring that statistical standards are maintained during the publication process.

Similar deficiencies in statistical methodology have also been reported across various biomedical disciplines. Previous methodological evaluations conducted in fields such as radiology, family medicine, dentistry, and psychology have consistently demonstrated that statistical errors and reporting deficiencies remain common in biomedical publications. For example, studies examining radiology journals have shown that a substantial proportion of published articles contain at least one statistical error, particularly in the reporting of descriptive statistics and *P*. Similarly, cross-sectional analyses of family medicine research articles and evaluations of medical residency theses have identified frequent problems related to inappropriate statistical test selection, incomplete reporting of statistical methods, and inadequate presentation of results. These findings suggest that limitations in statistical reporting represent a widespread methodological challenge across biomedical research rather than a problem limited to a single specialty.^7^^,^^8^^,^^18^^25^^-^^28^

To our knowledge, this study provides one of the most recent comprehensive evaluations of statistical reporting practices in PM&R journals and offers contemporary empirical evidence comparing journals indexed in different citation databases.

This study has several strengths. First, it includes a relatively large sample of articles published across both international and national PM&R journals, allowing meaningful comparisons between indexing systems. Despite these strengths, several limitations should be acknowledged. First, although the articles were independently evaluated by two researchers using predefined criteria based on widely accepted reporting guidelines such as CONSORT, STROBE, and SAMPL, some degree of subjective judgment in the assessment of statistical reporting quality cannot be entirely excluded. Second, the study reflects a contemporary cross-sectional snapshot of PM&R publications within a defined publication period and therefore does not allow evaluation of long-term temporal trends in statistical reporting practices. Finally, while sample size calculation reporting was evaluated, the specific methodological approaches used for effect size determination were not systematically classified.

Improving statistical reporting quality in PM&R research may require not only stricter editorial policies but also broader access to statistical education resources and methodological support tools. Internationally recognized resources such as the SAMPL guidelines, G*Power software, and educational reviews on sample size determination and effect size interpretation may assist researchers in improving statistical planning and reporting practices.^29^^-^^31^

## Conclusion

In conclusion, this study demonstrates that statistical reporting deficiencies remain common in PM&R clinical research articles. Although SCI-E indexed journals generally showed better adherence to recommended statistical practices, important methodological shortcomings persist in both journal groups. Strengthening statistical training for researchers, promoting adherence to international reporting guidelines, and increasing editorial scrutiny of statistical methodology may substantially improve the methodological quality and reproducibility of future research.

## Figures and Tables

**Table 1.** Statistical Reporting Quality and Methodological Indicators in Evaluated Clinical Research Articles

Domain	Criterion	Yes n (%)	No n (%)	Not applicable n (%)
Descriptive statistics	Mean ± SD reported	403 (93.1)	16 (3.7)	14 (3.2)
	Median / IQR / min–max reported	205 (47.3)	171 (39.5)	57 (13.2)
Statistical test reporting	Statistical tests clearly defined	424 (97.9)	9 (2.1)	
	Incorrect statistical test name used	8 (1.8)	425 (98.2)	
	Unused statistical test reported	3 (0.7)	430 (99.3)	
	Incorrect statistical test applied	16 (3.7)	417 (96.3)	
*P* reporting	Closed-form *P* presentation	214 (49.4)	219 (50.6)	
	*P* inconsistency	9 (2.1)	424 (97.9)	
	*P* reporting error	48 (11.1)	385 (88.9)	
	Important *P* not reported	24 (5.5)	409 (94.5)	
	Statistical notation error	43 (9.9)	400 (90.1)	
	Inconsistency between text/table/figure	49 (11.3)	384 (88.7)	
Methodological practices	Appropriate normality assessment	315 (72.7)	86 (19.9)	32 (7.4)
	Sample size calculation performed	243 (56.1)	174 (40.2)	16 (3.7)
	Post-hoc analysis performed	138 (31.9)	40 (9.2)	255 (58.9)
	Multiple comparison correction	120 (27.7)	58 (13.4)	255 (58.9)
	Effect size reported	221 (51.0)	212 (49.0)	
Transparency indicators	Statistical software reported	407 (94.0)	26 (6.0)	
	Ethical approval statement	412 (95.1)	3 (0.7)	3 (0.7)
	Data confidentiality statement	330 (76.2)	69 (15.9)	19 (4.4)

IQR, interquartile range.

**Table 2.** Comparison of Statistical Reporting Practices Between SCI-E and TR Dizin Indexed Journals

Domain	Criterion	Status	SCI-E, n (%)	TR, n (%)	*χ*² (*df*)	*P*
Descriptive statistics	Mean ± SD reported	Yes / No	332 (92.7) /	71 (94.7) / 2 (2.7)	0.270 (1)	.604^a^
		Not applicable	12 (3.4)			
		No	14 (3.9)	2 (2.7)		
	Median / IQR / min–max reported	Yes/	169 (47.2) /	36 (48.0) / 6 (8.0)	0.771 (1)	.380^a^
		Not applicable	51 (14.2)			
		No	138 (38.5)	33 (44.0)		
Statistical test selection	Appropriate normality assessment	Yes	249 (69.6)	66 (88.0)	8.896 (1)	.003^a^
		No	80 (22.3)	6 (8.0)		
	Sample size calculation	Yes	194 (54.2)	49 (65.3)	3.425 (1)	.064^a^
		No	151 (42.2)	23 (30.7)		
	Post-hoc analysis performed	Yes	116 (32.4)	22 (29.3)	0.055 (1)	.814^a^
		No	33 (9.2)	7 (9.3)		
	Multiple comparison correction	Yes	98 (27.4)	15 (20.0)	5.420 (1)	.019^a^
		No	49 (13.7)	19 (25.3)		
	Effect size reported	Yes	190 (53.1)	27 (36.0)	8.210 (1)	.004^a^
		No	168 (46.9)	48 (64.0)		
Statistical test reporting errors	Statistical tests clearly defined	Yes	350 (97.8)	74 (98.7)		1.000^b^
		No	8 (2.2)	1 (1.3)		
	Incorrect statistical test name used	Yes	6 (1.7)	2 (2.7)		.632^b^
		No	352 (98.3)	73 (97.3)		
	Unused statistical test reported	Yes	3 (0.8)	0 (0.0)		1.000^b^
		No	355 (99.2)	75 (100.0)		
	Incorrect statistical test applied	Yes	13 (3.6)	3 (4.0)		.746^b^
		No	345 (96.4)	72 (96.0)		
*P*-value reporting errors	Closed-form *P*-value presentation	Yes	159 (44.4)	55 (73.3)	20.748 (1)	<.001^a^
		No	199 (55.6)	20 (26.7)		
	Important *P*-value not reported	Yes	18 (5.0)	6 (8.0)		.278^b^
		No	340 (95.0)	69 (92.0)		
	*P*-value reporting error	Yes	32 (8.9)	16 (21.3)	9.665 (1)	.002^a^
		No	326 (91.1)	59 (78.7)		
	*P*-value inconsistency	Yes	6 (1.7)	3 (4.0)		.192^b^
		No	352 (98.3)	72 (96.0)		
	Statistical notation error	Yes	36 (10.1)	7 (9.3)	0.036 (1)	.849^a^
		No	322 (89.9)	68 (90.7)		
	Inconsistency between text / table / figure	Yes	40 (11.2)	9 (12.0)	0.042 (1)	.837^a^
		No	318 (88.8)	66 (88.0)		
Transparency indicators	Ethical approval statement	Yes	343 (99.1)	69 (100)		1.000^b^
		No	3 (0.9)	0 (0)		
	Data confidentiality statement	Yes	294 (86.5)	36 (61.0)	21.675(1)	<.001
		No	46 (13.5)	23 (39.0)		

*χ*², chi-square statistic; IQR, interquartile range; SCI-E, Science Citation Index Expanded.

^a^Pearson chi-square test; ^b^Fisher’s exact test.
